# Preparation of Water Suspensions of Nanocalcite for Cultural Heritage Applications

**DOI:** 10.3390/nano8040254

**Published:** 2018-04-19

**Authors:** Maria-Beatrice Coltelli, Dario Paolucci, Valter Castelvetro, Sabrina Bianchi, Elisabeth Mascha, Luca Panariello, Cecilia Pesce, Johannes Weber, Andrea Lazzeri

**Affiliations:** 1 Inter University National Consortium of Materials Science and Technology (INSTM), Via Giusti 9, 50121 Florence, Italy; maria.beatrice.coltelli@unipi.it (M.-B.C.); dario.paolo06@gmail.com (D.P.); valter.castelvetro@unipi.it (V.C.); sabrinab@dcci.unipi.it (S.B.); 2 Department of Civil and Industrial Engineering, University of Pisa, Via Diotisalvi 2, 56126 Pisa, Italy; luca.panariello@ing.unipi.it; 3 Department of Chemistry and Industrial Chemistry, University of Pisa, Via Giuseppe Moruzzi, 13, 56124 Pisa, Italy; 4 Institute of Arts and Technology/Conservation Sciences (IACTS), University of Applied Arts, Salzgries 14/1, A-1010 Wien, Austria; elisabeth.mascha@uni-ak.ac.at (E.M.); cecilia.pesce@uni-ak.ac.at (C.P.); johannes.weber@uni-ak.ac.at (J.W.)

**Keywords:** nanocalcite, heritage conservation, calcium carbonate, aqueous nanoparticles, consolidation

## Abstract

The consolidation of degraded carbonate stone used in ancient monuments is an important topic for European cultural heritage conservation. The products most frequently used as consolidants are based on tetraalkoxy- or alkylalkoxy-silanes (in particular tetraethyl-orthosilicate, TEOS), resulting in the formation of relatively stable amorphous silica or alkylated (hydrophobic) silica inside the stone pores. However, silica is not chemically compatible with carbonate stones; in this respect, nanocalcite may be a suitable alternative. The present work concerns the preparation of water suspensions of calcite nanoparticles (CCNPs) by controlled carbonation of slaked lime using a pilot-scale reactor. A simplified design of experiment was adopted for product optimization. Calcite nanoparticles of narrow size distribution averaging about 30 nm were successfully obtained, the concentration of the interfacial agent and the size of CaO being the most critical parameters. Primary nanoparticle aggregation causing flocculation could be substantially prevented by the addition of polymeric dispersants. Copolymer-based dispersants were produced in situ by controlled heterophase polymerisation mediated by an amphiphilic macro-RAFT (reversible addition-fragmentation transfer) agent. The stabilized CCNP aqueous dispersions were then applied on carbonate and silicate substrates; Scanning Electron Microscopy (SEM)analysis of cross-sections allowed the evaluation of pore penetration, interfacial binding, and bridging (gap-filling) properties of these novel consolidants.

## 1. Introduction

Designing new products suitable for conservation treatments of valuable cultural heritage objects and buildings is an open issue, due to the many requirements that such products should fulfil and the broad range of different materials to be treated. In the case of ancient stone materials, chemical and physical alteration due to natural weathering, often exacerbated by anthropogenic pollution, eventually results in deterioration phenomena lowering the stone cohesivity (microscopic) and mechanical properties (macroscopic). In such cases, the application of consolidants is a common practice. Unfortunately, commercially available consolidating products present various drawbacks related either to their performance or to undesired effects associated with their use. In fact, in addition to effectively rebuild mechanical strength, a consolidant should exhibit good compatibility with the treated stone and grant adequate durability, without damage caused by derivatives generated upon ageing (e.g., yellowing, discoloration, hydrolysis by acidic or alkaline by-products).

Among the numerous inorganic, polymeric, and hybrid materials that have been used in stone conservation, consolidant formulations based on tetraethoxysilane (or tetraethyl-orthosilicate, TEOS) are possibly the most common. Their effectiveness is the result of good penetration within the porous stone network and the good film-forming properties of TEOS, leading to the formation of relatively stable amorphous silica [1,2]. However, the syneresis of silane-based treatments upon drying and sol-gel condensation into silica-like material, or later on, due to the different thermal expansion behavior with respect to the host stone, easily results in microcracking, while incomplete condensation of hydrolyzed silanols leads to formation of a highly hydrophilic material. Furthermore, it would be desirable to employ a carbonate-based consolidant for treating a carbonate stone.

More recently, nanoparticles have been proposed as promising consolidation materials. If suitably designed, they may effectively penetrate the stone porosities and fill up the larger gaps generated by pore corrosion; once combined with film-forming materials in nanoparticle-modified formulations, they are expected to prevent or minimize the abovementioned problems of microfracturing and hygroscopicity. Silica nanoparticles have been reported to improve the mechanical resistance of a bioclastic sandstone calcarenite [3]; in combination with nanotitania, they result in self-cleaning stone surfaces [4]. In any case, both TEOS and nanosilica treatments are based on silica, which is clearly poorly compatible with carbonate stones. Intermediate solutions have been proposed, to combine the good adhesivity and penetration ability of silane-based consolidants with the stability and better affinity of inorganic materials. Formulations based on TEOS and nanocalcium oxalate precipitated in situ from calcium hydroxide and oxalic acid were reported to show improved compatibility with respect to TEOS [5,6]. The application of diammonium hydrogen phosphate to calcitic substrates for in situ generation of hydroxyapatite has been reported to improve the mechanical strength of porous carbonate stones [7,8]; however, not only hydroxyapatite, but also other metastable phosphate phases are formed, and small unreacted phosphate fractions remain in the stone, making necessary a second application of limewater to convert the excess phosphate. Alcoholic nanocalcium hydroxide has been used in consolidant and deacidification treatments of stones, wall paintings, paper, and wood [9,10,11]. The presence of alcohol was reported to favor the formation of calcium alkoxide, leading to the formation of a calcium carbonate polymorph rich in vaterite [12]; in fact, when calcium alkoxides were used as precursors, the crystalline structure of the obtained calcium carbonate was found to be dependent on the type of alcoholic solvent and dispersant [13]. Water-soluble calcium acetoacetate has also been explored as calcite precursor [14]. However, highly reactive treatments may result in poor penetration in small pores. Therefore, for the consolidation of a carbonate-containing stone, an aqueous dispersion of preformed calcite nanoparticles may be the best option due to the small aspect ratio (spheroidal shape of rhombohedral crystals), higher stability, and affinity with carbonate mineral. The nanometric size allows easy penetration of stone microporosities, but also effective gap filling of larger pores, while the large surface area promotes the aggregation and bridging of nanoparticles deposited inside the pores, due to electrostatic and van der Waals forces [15,16]. Thus, NPs, as a consequence of drying-mediated assembly [17,18], form a coherent material with a not negligible strength [19] inside the pores. The mechanism of adhesion involving NPs has not been investigated systematically up to now. In particular, adhesion between NPs and stone was never studied, whereas the increase in strength was verified in concrete [20] and mortars [21] treated with nanosilica. In mortars, the strength was found to increase with decreasing particle size up to 40 nm [21], in agreement with the expected increase of interfacial interactions of the NPs with the inorganic gel matrix. As in general, the bulk density of interaction is enhanced by a high surface-to-volume ratio, calcium carbonate NPs were expected to show a good adhesion to limestone because of both large surface area and high chemical affinity. Calcium carbonate particles can be produced by either top-down (mechanical milling) or bottom-up (chemical synthesis) routes [22,23]. The mechanical route requires long processing times to reduce the particle sizes below 1 μm, and a polymorph mixture (calcite, aragonite, and vaterite) is typically obtained. The chemical route is generally advantageous as it is faster, involves lower energy consumption with simple equipment, and allows better product control. Calcium carbonate nanoparticles (CCNPs) with average size in the 17–100 nm range have been synthesized by reactive precipitation in a high-pressure jet homogenizer [24], flame spraying [25], mechanochemical method [26], high-gravity reactive precipitation [27], microemulsion, and carbonation processes in a loop reactor [28]; the processes based on precipitation being the most frequently used because of their high yield, simplicity, and scalability. Lime carbonation by CO_2_, the most common industrial process for the production of CaCO_3_ powders, may be designed for the production of nanosized particles once the difficulty of controlling particle size and shape of CCNPs has been overcome [29]. For this purpose, the role of added surfactants [30], process temperature [31], and polymeric additives [32] on the controlled precipitation of CCNPs from highly supersaturated solutions have been investigated. CCNPs have been investigated as reinforcing material in polymer nanocomposites and concrete [33,34]. The toxicity of CaCO_3_ nanoparticles was also tested and found similar to that of conventional micrometric calcite [35].

The main objective of the present work was the design and investigation of an innovative gas–liquid carbonation process for the upscalable production of calcium carbonate nanoparticles with small size (less than 50 nm) and well-defined morphology (narrow size distribution, uniform shape, and well-defined crystal structure) from an aqueous slaked lime precursor in a pilot-scale batch reactor. In fact, such features are expected to play a crucial role for the effectiveness of stone consolidation treatments based on CCNPs. A distinctive feature of the process is the use of citric acid as a surface agent to control particle size and selectively obtain calcite among the calcium carbonate polymorphs. In fact, mono- and polycarboxylic acids are known to act as crystallization inhibitors in the initial stages of the carbonation process [36,37,38,39,40], the latter types promoting the growth of spherical nanocalcite particles.

A simplified design of experiment (DoE) was adopted for product optimization [41]. DoE may be an effective tool for the prediction of the properties of complex formulations [42], but also for the optimization of formulation and/or operational parameters in a synthetic process. In particular, four factors were selected as the reaction variables: average particle size of the CaO precursor, concentration of the interfacial agent, stirring rate, and CO_2_ flow rate.

Subsequently, the effectiveness of amphiphilic or double-hydrophilic acrylic copolymers as colloidal stabilizers of the aqueous CCNPs was investigated. Polycarboxylic copolymers are known to effectively control CaCO_3_ crystal growth [32,43] and stabilize CaCO_3_ dispersions, even in organic solvents [44]. In a previous work, a triblock copolymer of acrylic acid (AA) and oligoethylene glycol methyl ether methacrylate (OEGMA) was found to be an effective stabilizer, even for gold nanoparticles [45]. In this work, four different copolymers based on the same structural elements (acrylic acid and oligoethylene glycol segments), but with different structure (random and block, copolymers) and topology (linear and comb-like) were tested as colloidal CCNPs stabilizers. Preliminary application tests were finally performed to study the penetration effectiveness of CCNPs and their behavior once absorbed into porous carbonate and silicate stones, respectively.

## 2. Materials and Methods

### 2.1. Materials

Two grades of calcium oxide (CaO) powders were kindly provided by Unicalce S.p.A., Campiglia Marittima, Italy, and used as received. The raw materials differ for the average particle size (about 100 μm for the cheapest and most common one; about 30 μm for the micronized second one). The available lime in the starting CaO powder was determined by the ASTM C25-90 standard method.

Citric acid (99% ACS grade, Sigma Aldrich, Saint Louis, MO, USA) was used as received. De-ionized water (resistivity 18 MΩ) was produced with a mod. Osmodemi combined reverse osmosis/ionic exchange apparatus (Idrotecnica, Genua, Italy). Cylinders with a 70:30 N_2_/CO_2_ mixture were purchased by Sol Group S.p.A. (Milano, Italy).

### 2.2. Syntheses

Calcium carbonate nanoparticles (CCNPs) were obtained via carbonation precipitation from a slaked lime (hydrated calcium oxide) aqueous slurry. Two grades of commercial calcium oxide powders of different nominal particle size (100 and 30 μm, respectively) were tested. In order to investigate the influence of the CaO hydration process (lime slaking) on the morphology and size of the final CaCO_3_ particles, in preliminary experiments, the average Ca(OH)_2_ particle size and size distribution was measured as a function of hydration time and process conditions. In fact, it has been reported that several factors in the lime hydration process affect the specific surface of the calcium hydroxide; among them, the calcination process to manufacture CaO, the lime-to-water ratio, the slaking time, and temperature [46,47]. According to the general procedure, the CaO powder was sieved at 60 mesh, then 0.446 mol/L (25 g/L) CaO was slaked in a pilot-scale 10 liter jacketed cylindrical stainless steel reactor (50 cm height, 18 cm internal diameter, three 15 × 510 mm^2^ baffles soldered to the reactor wall) fitted with a pitched-blade turbine (four rectangular 20-mm long blades with 30° tilt) for mechanical stirring and equipped with a pH meter (CronospHSense, Process Instruments Ltd., Burnley, UK) and thermocouple. Slaking was performed by adding the CaO powder into a solution of citric acid (0.1–5 wt % of CaO) in 10 L deionized water. The slurry was stirred for fifteen minutes to allow conversion of the quicklime in slaked lime, then the valve connecting the CO_2_ line to the bottom of the reactor was opened and the slurry was purged under vigorous mechanical stirring while the temperature was maintained at 10 °C by means of the cooling fluid (1:1 ethylene glycol/water) circulated in the cooling jacket by a 1.5 kW-powered chiller. The CO_2_ flow rate was controlled by a gas flow meter, while an online pH meter was used to monitor the decrease from pH ~ 13 of the initial Ca(OH)_2_ slurry to a final pH ~ 7, when the CO_2_ flow was stopped since the carbonation reaction could be considered as completed.

### 2.3. Experimental Design

In order to obtain calcite nanoparticles with very small (<50 nm) and uniform size, a design of experiment (DoE) approach and ANOVA statistical analysis of the impact factors of the chosen process and formulation parameters was adopted. The objective of a design of experiments (DoE) is to establish a cause-and-effect relationship among a number of independent variables (factors) and a dependent variable of interest (response). In our case, the response was the average CaCO_3_ particle size, the process and formulation variables being the factors. In a typical setup, experiments are run at different factor values (levels). Out of the n^k^ possible experimental combinations (k = number of factors, n = number of levels, if the same number of levels is considered for each factor), that is, in a full factorial design, the DoE procedure allows the selection of a fractional factorial design (FFDoE) as to reduce the total number of experimental runs to n^k−1^ experimental combinations without significant loss of information that may be useful for process optimization. In the present study, two levels were considered for each one of the four selected factors for the synthesis of CCNPs, as follows: (i) 800 and 1200 m^3^/h for the CO_2_ flow rate; (ii) 800 and 1600 rpm for the stirring rate; (iii) 30 and 100 μm for the nominal particle size of the CaO precursor; and (iv) 1 and 5 wt % of citric acid with respect to the starting CaO. The resulting 16 possible combinations were then reduced to only 8 experiments according to a FFDoE. For the selection of the relevant experiments, the impact factor parameters, indicating how much the variation of a single factor affects the response, were analyzed and their validity assessed by an ANOVA (analysis of variance) method using the Minitab (Pennsylvania State University) software. The responses measured from the selected FFDoE set were analyzed graphically to assess the magnitude and importance of each factor by main effects plot and interaction plot. Based on the responses of the first set of experiments, suggesting that the 100-μm CaO particle size was a better option irrespective of the other factors, in a second optimization scheme, a simplified DoE procedure based on a cube plot was adopted. The cube plot is a graphical representation of a three factor–two level scheme corresponding to a maximum of eight further experiments in which only three factors are retained.

### 2.4. Characterization of Nanostructured Suspensions

A Micromeritics Sedigraph III 5120 Analyzer was used for the evaluation of the particle size of the as-prepared aqueous dispersions of calcium carbonate, while a Brookhaven 90Plus dynamic light scattering instrument equipped with a 35 mW Hamamatsu HC120 laser and Avalanche photodiode detector was used for particle sizing of polymer-stabilized nanocalcite. The actual size and shape of primary calcium carbonate particles was determined by scanning electron microscopy using a JEOL-JSM 5600LV and FEI Quanta 450 ESEM FEG environmental field emission instrument. Infrared spectra were recorded in the 550–4000 cm^−1^ range with a Nicolet 380 Thermo Corporation Fourier transform infrared (FTIR) spectrometer equipped with a smart Itx Attenuated Total Reflectance (ATR) accessory, collecting 256 scans at 4 cm^−1^ resolution. Crystalline phase type and fraction as well as average crystallite size of the prepared CaCO_3_ were determined with a Bruker D2 Phaser X-ray diffraction (XRD) instrument using Cu Kα radiation within the 4 < 2θ < 65 deg range, operating at 0.020 deg step displacement and 65.6 s step time. Diffraction patterns were compared with Joint Committee on Powder Diffraction Standards (JCPDS) standards. Specific surface area of dry nanoparticle powders was obtained from the adsorption isotherms according to the Brunauer-Emmett-Teller (BET) method using a Micromeritics Gemini V surface area analyzer operating with N_2_ as the adsorbate; standard outgassing pretreatment was 3 h at 250 °C under a vacuum. Thermogravimetric analyses for the calcium carbonate content were performed on 4–20 mg of sample using a Mettler-Toledo Thermogravimetric Analysis/Scanning Differential Thermal Analysis (TGA/SDTA) 851 instrument operating with nitrogen as the purge gas (60 mL/min) at 10 °C/min heating rate in the 25–1000 °C temperature range.

### 2.5. Colloidal Stabilizers of the Nanocalcite Suspensions

For the stabilization of the precipitated CCNPs, dispersions of four different acrylic acid copolymers with oligoethylene glycol segments either in the main chain (linear structure) or in the side chain (comb-like) were tested. The first one, hereafter PCA1, is a poly(acrylic acid-*co*-polyethylene glycol methyl ether methacrylate) (poly(AA-*r*-PEGMA)) of commercial source (developmental comb-like copolymer from BASF). The other three, synthetized in this work, are: (i) a random copolymer of acrylic acid (AA), poly(acrylic acid-*co*-oligoethylene glycol methyl ether methacrylate), poly(AA-*r*-OEGMA), with similar comb-like structure to the previous one, the main differences being a higher degree of polymerization (average number of monomer units per chain) and the shorter methoxy-terminated oligoethylene glycol ester chain of OEGMA with ~8 ethylene glycol (EG) units; (ii) a comb-like triblock copolymer, poly(OEGMA-*b*-AA-*b*-OEGMA); (iii) a linear block copolymer, poly(AA-*b*-MPEG), where MPEG is the monomethylether of a polyethylene glycol with about 17 EG units (MW ~ 750 g/mol). The latter three copolymers were synthesized by RAFT polymerization in an aqueous medium, using either a symmetric (difunctional) or an asymmetric (monofunctional) trithiocarbonate. In particular, the difunctional *S*,*S*’-bis(α,α’-dimethyl-α’’-acetic acid)-trithiocarbonate (TTdCA) was used as the RAFT mediator for the synthesis of the random and block AA/OEGMA comb-like copolymers, respectively; while the monofunctional 2-(dodecylthiocarbonothioylthio)-2-methylpropanoic acid (TTCA) was firstly modified with MPEG by direct Steglich esterification in the presence of dicyclohexylcarbodiimide 4-dimethylaminopyridine coupling agent and catalyst to obtain the amphiphilic MPEG-TTCA [48], and then extended with AA by RAFT polymerization to obtain poly(AA-*b*-MPEG). The trithiocarbonates TTdCA and TTCA were synthesized as reported by Lai [49].

### 2.6. Preliminary Test Treatments on Porous Substrates

**Figure 1:**
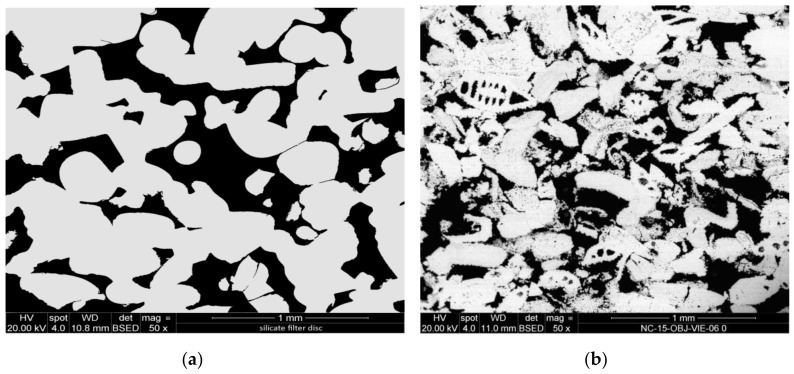
SEM-backscattered electron (BSE) micrographs of the cross-sections of: (**a**) silicate glass disc; (**b**) Au detrital limestone.

For a preliminary evaluation, the different CCNP aqueous dispersions were applied onto two reference porous substrates: a synthetic silicate glass (20 mm diameter/2 mm thick Duran^®^ P1 filter disc, nominal pore size 100–160 µm) and a natural carbonate (“Au” limestone, Austria; a calcitic biosparite grainstone with grain size up to 1.2 mm showing interparticle, intraparticle, and dissolution pores; 10 × 10 × 10 mm^3^ cubic specimen). In fact, silicate filter discs have successfully been used as model porous substrates for preliminary screening of consolidant effectiveness [50], since the chemical homogeneity of the material and the range of well-defined pore sizes allows a detailed evaluation of the distribution of the applied consolidant (Figure 1a). For the same reasons, freshly quarried Au limestone (Figure 1b) may be regarded as a reference calcareous substrate, in addition to being a building material of high historic interest (extensively used in Vienna, e.g., in the St. Stephan Cathedral and throughout the middle age until the second half of the 19th century) [51], and having for this reason been among the stone materials tested in a presently running European Project [52]. The penetration depth and distribution of dry material within the pores, the quality of the adhesion of the consolidant to the substrate, the cohesivity, and bridging capacity of the consolidant within the pores were assessed by scanning electron microscopy (SEM) analysis.

### 2.7. Stone Sample Treatment and Characterization

Before the application test, the natural Au limestone specimens were rinsed with water and gently brushed, then dried at 50 °C in an oven to constant weight; the silicate filter discs were used as received. Application of the CCNPs as 5 wt % aqueous dispersions was performed either by rising capillary suction (Au limestone) or by impregnation upon pipette dripping onto the top surface (silicate filter discs, its larger pores resulting in lower capillary pressure). The impregnation was continued until full saturation of the substrate, or for a maximum 1 h. The procedure was repeated after 24 h on the specimens partially dried under lab conditions, then the specimens were allowed to dry under lab conditions until constant weight, which always occurred within 3 days. For the SEM analyses, the dried specimens were vacuum-embedded in epoxy resin and polished cross sections were produced. A Philips XL 30 ESEM instrument equipped with a back-scattered electron detector (BSE) and an energy-dispersive X-ray analyzer (Link-ISIS), operated at 20 kV under low vacuum, was used to collect the SEM micrographs.

## 3. Results

The general procedure for the synthesis of calcium carbonate nanoparticles (CCNPs) by carbonation of slaked lime was optimized by considering two compositional (concentration of the citric acid additive, granulometry of the starting calcium oxide powder) and two process (CO_2_ flow rate, stirring rate) parameters, which were varied in order to obtain small (i.e., less than 50 nm) nanocalcite particles with narrow size distribution and uniform morphology, possibly resulting in aqueous dispersions with adequate colloidal stability. Citric acid was introduced based on previous experimental trials that had shown its effectiveness as an additive influencing both the nucleation and the stabilization of the precipitated calcite particles.

### 3.1. Influence of Process Parameters on CCNP Size and Morphology

In order to obtain calcite nanoparticles with very small size, as required for applications in which the aqueous nanoparticle dispersion must penetrate efficiently into natural stones of different chemical composition and pore structure, k = 4 experimental parameters were considered as potentially critical and thus worth investigating. As a means to reduce the number of synthetic runs potentially required to exhaustively explore the multidimensional experimental space generated by the k experimental parameters and their respective variability range, a design of experiment (DoE) approach and ANOVA statistical analysis of the impact of the chosen process and formulation parameters (factors) was adopted. In the simplest two-level (n = 2 chosen values for each parameter) experimental plan, a full factorial design would require a data set of n^k^ = 16 experimental runs to be performed, plus additional centerpoint runs including intermediate values between the two levels for one or more factors. As detailed in the experimental part, by adopting a fractional factorial DoE (FFDoE), the data set was initially reduced to the eight preparation trials listed as P1-P8 in Table 1. The result of the experiment, or response, is the particle size of the calcium carbonate particles, which was measured by photon correlation spectroscopy from dynamic light scattering (DLS) of the aqueous dispersions and by scanning electron microscopy (SEM) of the dried particles. The experimental results were analyzed with the Minitab^®^ software; the results from the fitted model and graphs allow the determination of which are the most important factors; that is, those that most effectively lead to a reduction of the calcium carbonate particles’ size. In particular, the software was used to fit the fractional factorial model, which includes the main effects and the two-way interactions.

**Figure 2:**
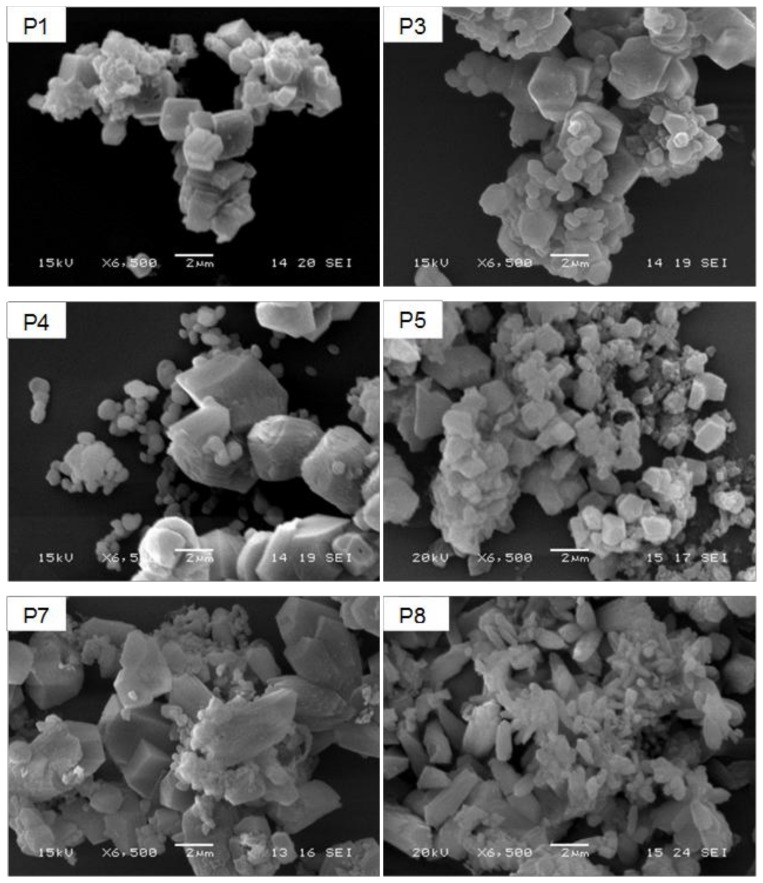
SEM micrographs of micrometer-sized precipitated CaCO_3_ particles as obtained from the P1, P3, P4; P5, P7 and P8 preparation trials.

**Figure 3:**
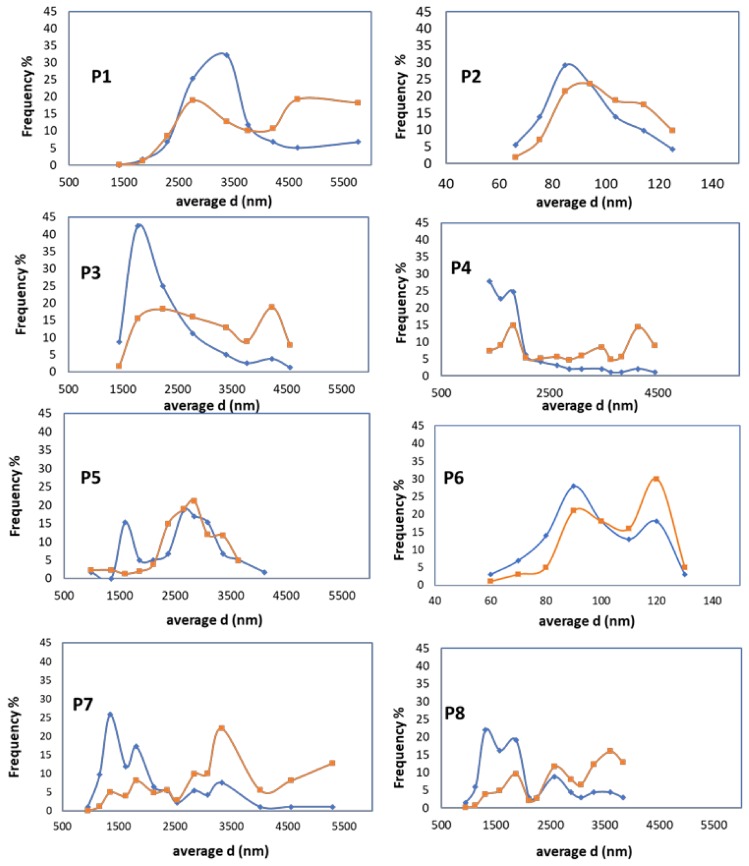
Calcium carbonate particle diameter (d) for samples P1–P8: number average (blue line) and weight average (orange line) distribution.

Figure 2 shows SEM micrographs taken from representative samples of precipitated calcium carbonate, which were image-processed to evaluate the number-average and volume-average particle size. All samples appear as aggregates of primary crystals, with clear differences in both the morphology and size of the primary particles. In particular, those obtained starting from the smaller sized 30-μm CaO powder (runs P1, P3, P5, and P7) are mainly composed of micrometer-sized rhombohedral crystals, probably originating from intergrown nucleated crystals. On the other hand, among those obtained starting from the larger sized 100-μm CaO powder, samples P4 and P8 show crystals still relatively large but with broader and bimodal size distribution (as it is better highlighted in the size distribution curves of Figure 3) and a progressive transition from the rhombohedral (P4) to a more elongated scalenohedral habit (P8), both typical of the calcite polymorph.

**Figure 4:**
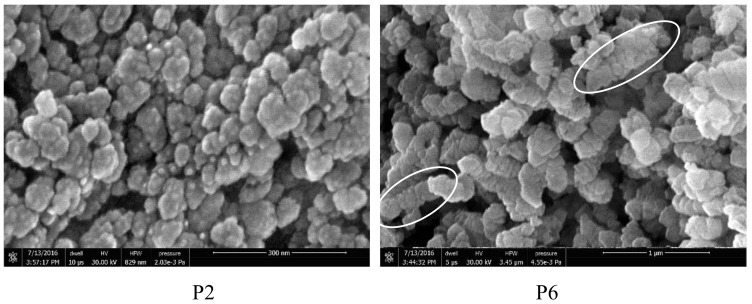
Field Emission (FE) SEM micrographs of the products from runs P2 and P6. Circles indicate “chain-like” submicrometric aggregates.

Finally, samples P2 and P6, obtained using the larger sized CaO powder and a low amount (1 wt %) of citric acid additive, show a distinctive nanosized morphology of the primary crystals. These, in addition to being much smaller in size than those from the other runs, show a pseudospherical habit and are arranged in “chain-like” submicrometric aggregates about 400–500 nm long (Figure 4).

Compared to the synthetic conditions reported previously [23], in which no additive was used, in the present case, the addition of citrate acting as a template during the precipitation allowed the obtainment of CCNPs from a larger sized and not thermally pretreated CaO powder (100 μm compared to <20 μm) using a lower CO_2_ flow rate (1.7 × 10^−4^ Nm^3^/s for a 10 L slurry, i.e., 0.017 L_CO_2__·L_CaO(aq)_^−1^·s^−1^, compared to 0.042 L_CO_2__·L_CaO(aq)_^−1^·s^−1^).

The number- and weight-averaged size calculated from the size distribution curves obtained from the SEM micrographs are reported in Table 1. The size distribution graphs in Figure 3, however, clearly show that all products but P2 and P6 are characterized by a broad and multimodal size distribution of the primary particles, with the largest volume fraction exceeding 2000 nm.

**Figure 5:**
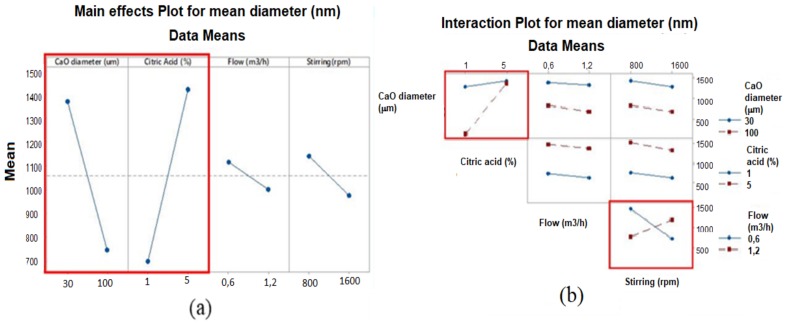
ANOVA graphs about effect of parameters: (**a**) main effect plot for mean diameter; (**b**) interaction plot.

ANOVA was used to assess the statistical significance of the differences among the means of several populations. In Figure 5 are reported the main effect plot and the interaction plot obtained using the Minitab statistical analysis software. In our case, two factors, the size of the CaO powder and the citric acid concentration, as well as their product (interaction between the two factors), can be considered as statistically significant.

Factorial plots illustrate how much a response is affected by one or more factors. As shown in Figure 5a, the main effect plots highlight the difference in the mean response as a result of the variation between two levels of a single factor. Each point represents the mean particle size for a given level of a factor; hence, if the line is not horizontal, a main effect is found, which means that different levels of the factor will affect the response differently. The steeper the line slope, the greater the magnitude of the main effect. The two highlighted effects, CaO powder size and citric acid concentration, were found to positively affect CCNP mean size.

Since the interactions are significant, the interaction plot should also be examined. A significant interaction between two factors can affect the interpretation of the main effects. The interaction plots of Figure 5b clearly show how the response (CCNP mean size) varies with the variation of one factor’s level while the level of a second factor is kept constant. The greater the difference in slope between the two lines in the same graph, the stronger the interaction. Among all the secondary interaction plots obtained, the graphs of Figure 5b that indicate a statistically significant interaction are: [CaO particle size]/[citric acid concentration] and [CO_2_ flow]/[stirring rate]. However, only the former interaction includes the factors that positively affect the primary CaCO_3_ particle size within the levels selected for the four factors in the first set of experiments.

**Figure 6:**
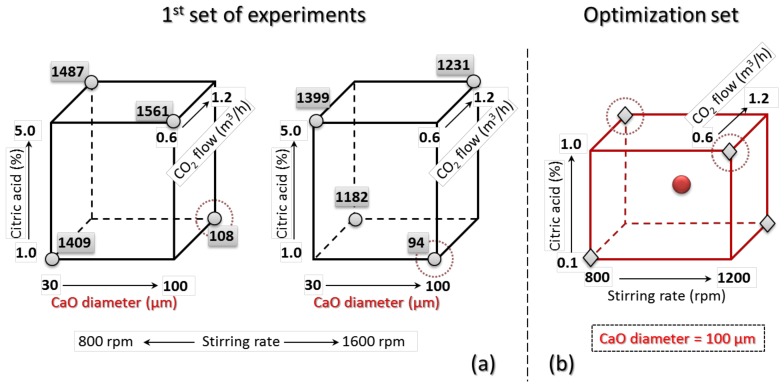
Cube plots for the DoE: each dimension of the 3D plot represents a different factor, with the selected two levels at the vertices. In (**a**), the numbers at the corners are the responses (mean size of calcite particles) from the first FFDoE set (the two cube plots differ only for the level of the fourth factor, the stirring rate); (**b**) optimized FFDoE with highlighted second set of five experimental runs, comprising one for each factor/level combination (diamonds) and one additional mean run (circle); the dotted circles refer to run P10 and P13 in the optimized set (see Table 2), corresponding to runs P6 and P2 of the first FFDoE, respectively; that is, those that gave the two best results.

For the optimization of the product, aiming at a further reduction of the mean size of the CCNPs, the responses of the first reduced set as depicted in the cube plots of Figure 6a were analyzed to design a second set of experiments. The three axes of the cube plot represent three of the four factors, with the two respective selected levels at the vertices of each edge; the fourth factor is displayed through a twin plot differing from the first one only for the different stirring rate. The numbers at the vertices indicate the response (average size of the CCNPs) from the corresponding experiment of the planned eight in the reduced first set.

Since the best products in the first set of experiments, P2 and P6, were both obtained using the larger sized CaO powder and the lower citric acid concentration, a reasonable assumption is that such a combination of values (corresponding to the lower left edges of the two cube plots in Figure 6a represents a starting point for further optimization; this would reduce the number of factors to be considered. On the other hand, the low importance (negligible effect on the response) of the other two parameters, CO_2_ flow and stirring rate, was quite surprising, suggesting that the optimal set of values for the four factors considered here might lie outside of the explored range of levels for at least one of the factors.

Based on previous (unreported) tests and on the known activity of citric acid as a chelating agent and inhibitor of calcite nucleation at relatively high concentrations [53], a second set of experiments was thus designed, in which the levels of citric acid concentration were lowered to the 0.1–1.0 range, while only the 100-μm CaO powder was considered, thus reducing the number of factors from four to three. Following the same FFDoE approach, a second set of five experiments was defined as reported graphically in the cube plot of Figure 6b and detailed in Table 2.

**Figure 7:**
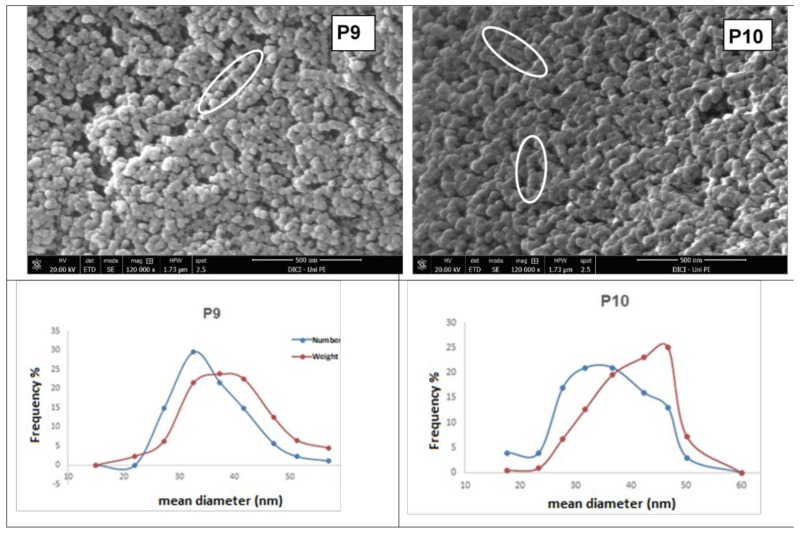
Above: SEM micrographs of dried powders from samples P9 (**left**) and P10 (**right**) with the corresponding particle size distributions below (the red line connects the weight-averaged frequency, the blue line the volume averaged-frequency) from particle counts and sizing obtained through image analysis. Circles evidence “chain-like” assemblies of nanoparticles.

All the experiments of this second set gave milky dispersions of CCNPs with similar short-term colloidal stability, followed by reversible flocculation. Representative Field Emission SEM (FE-SEM) micrographs of the products from runs P9 and P10 are shown in Figure 7, along with the particle size distribution curves obtained by image analysis. It is apparent that these latter products consist of small calcium carbonate primary particles (25–50 nm) with narrow size distribution. Chain-like assembly of nanoparticles was frequently observed during the SEM examination of these latter five products.

Thermogravimetric tests performed on all nanocalcite samples in nitrogen up to 1000 °C allowed determination of the yield of the conversion of calcium hydroxide to calcium carbonate from the mass loss occurring between 690 °C and 810 °C due to the loss of carbon dioxide. It was found that the conversion was almost complete, as the yield ranged between 94% and 99%.

The BET surface area of P9 dried powder was 42 m^2^/g. From a rough calculation, such a surface area yields a diameter of 53 nm for hypothetical monodisperse calcite nanospheres; this is in good agreement with the average of 33 nm determined by image analysis from the SEM micrographs of P9 dried powder, in which the nanoparticles are partially agglomerated (reversibly) and/or aggregated (irreversibly), with consequent reduction of the accessible surface area.

According to the Wide Angle X-Ray Diffraction (WAXD) analysis (data not shown), the crystal structure of the obtained CCNPs is almost 100% calcite, while the crystallite sizes, calculated according to the Debye-Scherrer equation from the width at half height of the most intense diffraction peak, are in good agreement with the Figure 4 and Figure 7; this applies in particular to samples P2, P9, and P10 (average sizes of 66, 34, and 31 nm, respectively), showing the smallest and most uniform crystallite size.

### 3.2. Stabilization of the Aqueous Nanocalcite Dispersions by Adsorption of Amphiphilic or Double-Hydrophilic Copolymers

**Figure 8:**
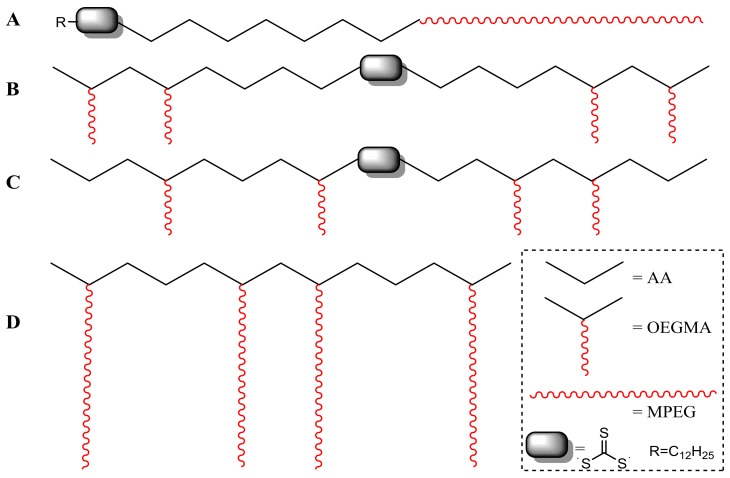
Polymer stabilizers used for the calcite nanoparticles (CCNPs): (**A**) poly(AA-*b*-MPEG); (**B**) poly(OEGMA-*b*-AA-*b*-OEGMA); (**C**) poly(AA-*r*-OEGMA); (**D**) PCA1, a developmental poly(AA-*r*-PEGMA) from BASF (Treviso, Italy).

All the dispersions showed a more or less pronounced colloidal instability, resulting in the sedimentation of larger calcite particles or flocculation of aggregates of smaller primary nanoparticles; the latter being already present in the as-synthesized product as shown by the DLS measurements. While the colloidal stability could be significantly improved through adjustment of the process parameters and feed formulation, a better long-term stability is required for practical product application and acceptance by the final users. For this purpose, four copolymers of acrylic acid (AA) comprising oligoethylene glycol segments either in the main chain (one linear block copolymer) or in the side chain (three comb-like copolymers) were tested as dispersing additives. The linear diblock copolymer poly(AA-*b*-MPEG) was prepared by controlled free-radical RAFT polymerization of AA mediated by the amphiphilic macro-RAFT agent MPEG-TTCA; the latter was obtained by esterification of monomethoxy oligoethylene glycol MPEG (~44 ethylene glycol units) with the asymmetric RAFT-active trithiocarbonate 2-(dodecylthiocarbonothioylthio)-2-methylpropanoic acid (TTCA). Two comb-like copolymers, a random poly(AA-*r*-OEGMA) and a double-hydrophilic triblock poly(OEGMA-*b*-AA-*b*-OEGMA), were prepared by RAFT polymerization of AA with the oligoethylene glycol methyl ether methacrylate OEGMA (~8 EG units), using the difunctional *S*,*S*’-bis(α,α’-dimethyl-α’’-acetic acid)-trithiocarbonate (TTdCA) as the RAFT agent. A third comb-like copolymer with longer polyethylene glycol side chains, poly(AA-*r*-PEGMA), was a developmental product of commercial source. A schematic illustration of the different copolymer structures is shown in Figure 8, while the characteristics of the copolymers, as determined by ^1^H-NMR, are listed in Table 3.

**Figure 9:**
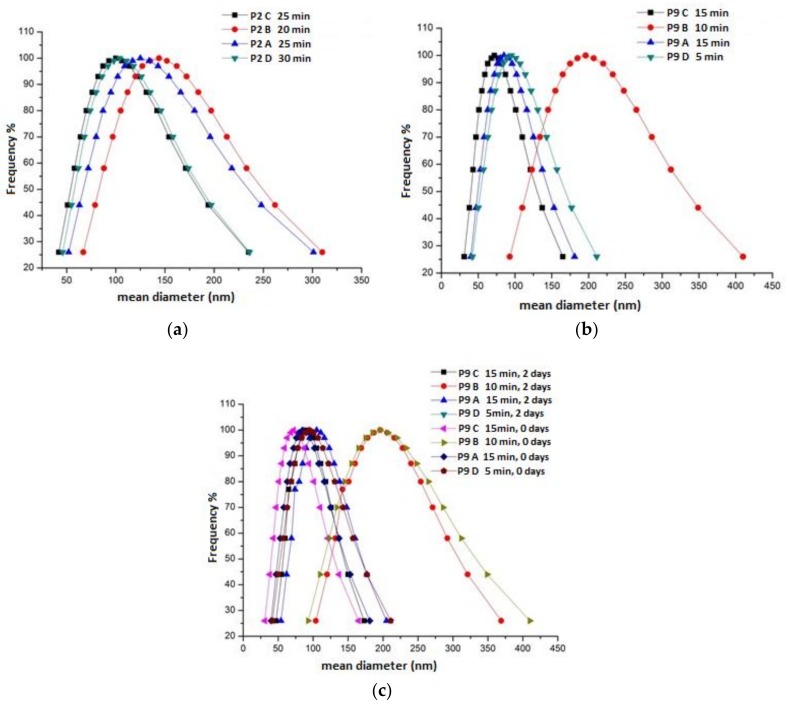
Particle size distribution curves of the optimized aqueous CCNP suspensions P2 and P9. Each curve shows the size distribution at the specified time elapsed after addition of the colloidal stabilizer: (**a**) sample P2; (**b**) sample P9; (**c**) sample P9 (two sets of measurements, right after and two days after sonication, respectively).

Colloidal stability tests were performed on products P2 and P9; that is, those actually consisting of nanometric primary calcite particles. For this purpose, 5 mL of each CCNP water dispersion was added to the given polymer stabilizer (5 wt % with respect to the dry CCNPs) and sonicated with a 3-mm tip diameter ultrasonic probe (dr. Hielscher, 400 W) for six 5 min cycles (0.5 s/cycle intermittence, 260 W/cm^2^ delivered power). After switching off the sonication, the dispersions were analyzed by DLS to monitor the average particle size as a function of time. In Figure 9a,b are reported the size distribution curves obtained by DLS from the polymer-modified P2 and P9 upon optimization of the sonication time. The minimum average size was measured after 5 to 30 min depending on the sample composition, further sonication generally resulting in worsening of the quality of the dispersions. Under the tested conditions, no significant improvement in the colloidal stability was observed by increasing the copolymer-to-CCNP weight ratio.

From the obtained particle size distribution curves, the most striking evidence is that while all the copolymers did improve the colloidal stability of the CCNPs, the three comb-like copolymers (B, C, and D) were the most effective in dispersing primary nanoparticles and smaller aggregates (typically less than 100 nm in size by DLS). In particular, the optimized product P9 was effectively stabilized even with the block copolymer B, a poorer performer than the two random copolymers, but still quite effective as compared with the linear block copolymer A. The effectiveness as nanoparticle dispersants of comb-like double-hydrophilic copolymers based on a poly(acrylic acid) (PAA) polyelectrolyte backbone and on polyethyleneoxide (PEO) side arms has been reported in the literature [54], and is ascribed to the combined contribution of the adsorption of the PAA main chain onto the particle surface (ostensibly quite effective in our case due to the formation of Ca^2+^ dicarboxylate) and to the ionic strength and pH-insensitive nonionic PEO teeth grafted onto the backbone and extending into the aqueous phase, thus providing steric stabilization to the colloidal particles. Concerning the observed better effectiveness of the random copolymer C compared to the parent block copolymer B, one can speculate that while block copolymers similar to B have been reported as somewhat effective micro- and nanoparticle dispersants [55], the presence of charge-neutral PEO teeth grafted at random intervals along the PAA backbone shall provide a more uniform steric screen for depletion stabilization of colloidal particles [56], and at the same time, effective shielding of the underlying PAA backbone from possible ion-bridging interactions that would lead to rapid aggregation of PAA-stabilized CCNPs in the presence of dissolved divalent Ca^2+^ ions.

Figure 9c shows that two days after the addition of the polymer stabilizers, the particle size distribution of sample P9 was nearly unchanged, confirming the improved colloidal stability of the dispersion. In fact, the CCNPs modified with either copolymer C or D showed substantial colloidal stability even after two weeks, the minimum shelf-life required for a product to be delivered to the application site where the consolidation procedure has to be carried out.

### 3.3. Pickup of Nanoparticle Dispersions by Porous Substrates and Penetration Depth

For both model substrates, the silicate filter discs and the Au limestone, progress of impregnation with the consolidant product, sample P9, could be checked visually from the advancement of the wetting front in the stone already during product application. Thereafter, the actual presence of the CCNPs within the porous network of both substrates was confirmed by SEM-BSE. However, while the presence of CCNPs was confirmed throughout the whole stone specimens, the in-depth distribution of the consolidant layer generated by the solid matter upon drying was found to be not homogeneous.

**Figure 10:**
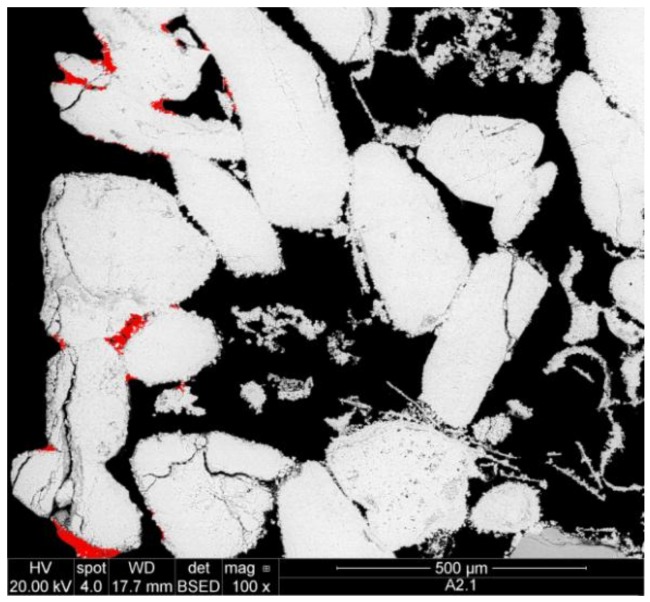
SEM-BSE of Au limestone with accumulation of consolidant (marked red) on the edges of the stone specimen.

In the case of the Au limestone, the highest amount of the precipitate (about 90%) was observed on the bottom and the sides of the specimen (Figure 10), while in the inner pore volume (deeper than 0.5 mm), only very small amounts of the solid consolidant could be detected. Similarly, in the case of the silicate filter discs, in which the product was applied from the top side, about 90% of CCNPs was accumulated in the upper 0.2 mm, and only small amounts of the consolidant could be traced in the interior and the bottom layer of the specimens.

Such uneven distribution might be due to various factors not necessarily related to the ratio between particle size and pore size/pore morphology, but also to the steric and electrostatic interactions between the CCNPs and the pore surfaces, leading to incipient agglomeration and precipitation during the impregnation. In such occurrences, the adsorbed copolymers used as stabilizers would most likely play an important role. Another possible cause could be a too-fast evaporation of the aqueous vehicle. In fact, a similar uneven distribution of the consolidant had also been found for treatments with nanolime [57], whereupon a successive thorough optimization of the application and curing procedure—not yet performed for nanocalcite, but undoubtedly worth investigating as a further development of the present research—had resulted in more uniform consolidant distribution.

**Figure 11:**
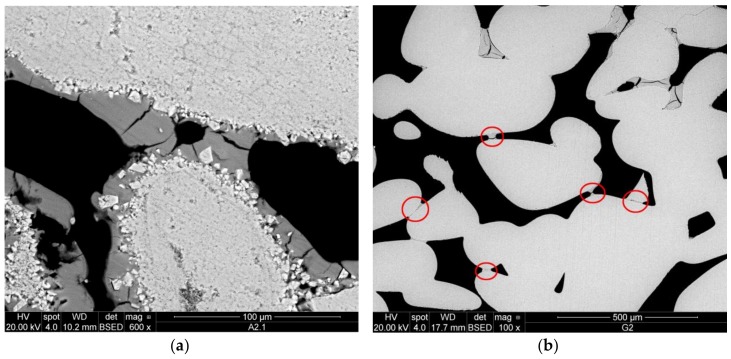
SEM-BSE micrographs showing: (**a**) very good adhesion and capacity to form bridges of nanocalcite on Au limestone; (**b**) silicate filter disc with small bridges of nanocalcite and moderate shrinkage properties of the product.

On the other hand, a more promising performance of the aqueous CCNPs as a stone consolidant could be highlighted when considering such critical properties as adhesion, shrinkage of the solid layer upon drying and stabilization, and the related bridging capacity with respect to the gaps between loosely interacting stone grains typically found in deteriorated stones. In fact, a very tight adhesion of nanocalcite to the carbonate grain surfaces of Au limestone (Figure 11a) was noticed. The product was able to form bridges, while only moderate shrinkage phenomena could be observed. 

In the silicate filter discs (Figure 11b), good adhesion of nanocalcite to the glass surfaces of the substrate was found in most of the interface. Shrinkage of the consolidant occurred mainly where accumulation of the product was observed, mainly near the top surface of the specimen. However, even if only small amounts of consolidant could be detected within the pores, small bridges of CCNPs were visible within the whole specimen.

## 4. Conclusions

A simple, cost-effective, and industrially scalable process for the preparation of aqueous dispersions of calcite nanoparticles by controlled carbonation of slaked calcium oxide using a semi-batch pilot reactor was successfully defined. The process conditions and feed composition were optimized using a FFDoE approach to achieve the reproducible synthesis of calcite nanoparticles of uniform morphology and narrow size distribution centered at 30 nm. The concentration of citric acid as a nucleation and interfacial agent turned out to be a critical parameter, along with the particle size of calcium oxide powder. Unexpectedly, the larger sized out of the two grades of commercial CaO powders used, without any thermal pretreatment, as the slaked lime precursors gave the smallest CCNPs. In order to further improve the colloidal stability of the optimized CCNP aqueous dispersions, a set of amphiphilic and double-hydrophilic acrylic acid copolymers containing PEG blocks either in the main chain (linear copolymers) or in the side chain (comb-like) were investigated as colloidal stabilizers. The double-hydrophilic comb-like copolymers with a random distribution of polyether side chains were found to be the most effective.

The effectiveness of the obtained nanocalcite suspensions as potential consolidants for deteriorated calcareous and siliceous stone substrates was investigated in preliminary experiments, in which the CCNP aqueous dispersions were applied onto model porous substrates. Stratigraphic SEM analyses have shown partial and irregular product penetration within the porous network, but also good adhesion of the precipitated CCNPs, as shown by the effective formation of bridges connecting stone grains. Shrinkage of the consolidating film upon drying was minor in the treated Au limestone and only moderate in the silicate samples. The uneven CCNP distribution throughout the porous stone network, along with local accumulation of the consolidant at the edges of the treated stone specimens, was ascribed to rapid evaporation of the treating solution; in order to overcome this problem, a more thorough investigation to define the best application protocol will be required. Furthermore, application tests to be performed on a broader range of natural stones and other porous substrates, as well additional ones to evaluate the effects on relevant physico-mechanical parameters, are still needed to fully assess the potential of this class of products as consolidants. It is worth pointing out that further improvements concerning both the CCNP production process and their colloidal stabilization or even encapsulation with suitably designed copolymers may allow further improvement of the elasticity and cohesivity of the nanoparticle-built bridges within the pores. The results obtained so far, while limited to the application on only two reference substrates, do not yet allow the anticipation of a significant short-term strengthening effect of the CCNPs, in particular because of the observed low penetration depth and the lack of scientific studies regarding the chemical bonding of calcite nanoparticles with either carbonate or siliceous substrates. Thus, while some reduction of the overall porosity could be achieved, proper mechanical strengthening still needs to be confirmed. However, further improvements could be realized by optimizing the application protocols (e.g., by adjusting the concentration of NPs in the formulation or by multiple application cycles) or by suitable modification of the CCNPs through nanoencapsulation with a thin polymer shell. The results of ongoing testing and further development of these newly designed CCNP-based consolidants will be the subject of a forthcoming paper.

## Figures and Tables

**Table 1:** Data set for the fractional factorial design of experiment (FFDoE). P1-8 are different preparation trials.

Run	Factors				Response
	CaO Particle Size ^(a)^ (μm)	Citric Acid (wt %)	CO_2_ Flow Rate (10^−4^ Nm^3^/s)	Stirring Rate (rps)	CaCO_3_ Particle Size ^(b)^ (nm)
P1	30	1	1.7	13.3	1409
P2	100	1	1.7	26.7	94
P3	30	5	1.7	26.7	1399
P4	100	5	1.7	13.3	1561
P5	30	1	3.3	26.7	1182
P6	100	1	3.3	13.3	108
P7	30	5	3.3	13.3	1487
P8	100	5	3.3	26.7	1231

^(a)^ Nominal size from technical sheet of the commercial product; CaO conc. 0.446 mol/L (25 g/L). ^(b)^ Volume average particle size by SEM.

**Table 2:** Data set for the optimization runs.

Run	Factors			Response
	Citric Acid (wt %)	CO_2_ Flow Rate (10^−4^ Nm^3^/s)	Stirring Rate (rps)	CaCO_3_ Particle Size ^(a)^ (nm)
P9	0.1	1.7	13.3	33
P10	1.0	3.3	13.3	33
P11	0.55	2.5	20	^(b)^
P12	0.1	3.3	26.7	^(b)^
P13	1.0	1.7	26.7	^(b)^

^(a)^ Mean diameter as determined by image analysis from SEM micrographs. ^(b)^ Reversible flocculation with short term colloidal stability (particle size not measured; similar appearance as in P9 and P10).

**Table 3:** Structural features of the copolymers used as colloidal stabilizers.

	Polymer	RAFT Agent	Feed Molar Composition	MW (g/mol)
A	poly(AA-*b*-MPEG)	TTCA	n.a.	3100
B	poly(OEGMA-*b*-AA-*b*-OEGMA)	TTdCA	AA/OEGMA_450_ = 3/1	23,500
C	poly(AA-*r*-OEGMA)	TTdCA	AA/OEGMA_450_ = 6.5/1	20,000
D	PCA1 ^(a)^	n.a.	AA/OEGMA_x_ = 2.5/1 ^(b)^	n.a.

^(a)^ BASF product; ^(b)^ Chemical composition provided by the supplier. n.a.: information not available.
